# Genetic diversity within a tree and alternative indexes for different evolutionary effects

**DOI:** 10.1017/qpb.2024.9

**Published:** 2024-12-06

**Authors:** Yoh Iwasa, Sou Tomimoto, Akiko Satake

**Affiliations:** 1 Department of Biology, Faculty of Science, Kyushu University, 744 Motooka, Nishi-ku, Fukuoka 819-0395, Japan; 2 Graduate School of Systems Life Sciences, Kyushu University, 744 Motooka, Nishi-ku, Fukuoka 819-0395, Japan

**Keywords:** modular organism, pairwise phylogenetic distance, parent-offspring distance, phylogenetic diversity, somatic mutations

## Abstract

Trees, living for centuries, accumulate somatic mutations in their growing trunks and branches, causing genetic divergence within a single tree. Stem cell lineages in a shoot apical meristem accumulate mutations independently and diverge from each other. In plants, somatic mutations can alter the genetic composition of reproductive organs and gametes, impacting future generations. To evaluate the genetic variation among a tree’s reproductive organs, we consider three indexes: mean pairwise phylogenetic distance ( 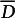 ), phylogenetic diversity ( 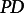 ; sum of branch lengths in molecular phylogeny) and parent-offspring phylogenetic distance ( 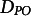 ). The tissue architecture of trees facilitated the accumulation of somatic mutations, which have various evolutionary effects, including enhancing fitness under strong sib competition and intense host-pathogen interactions, efficiently eliminating deleterious mutations through epistasis and increasing genetic variance in the population. Choosing appropriate indexes for the genetic diversity of somatic mutations depends on the specific aspect of evolutionary influence being assessed.

## Graphical abstract

**Figure:**
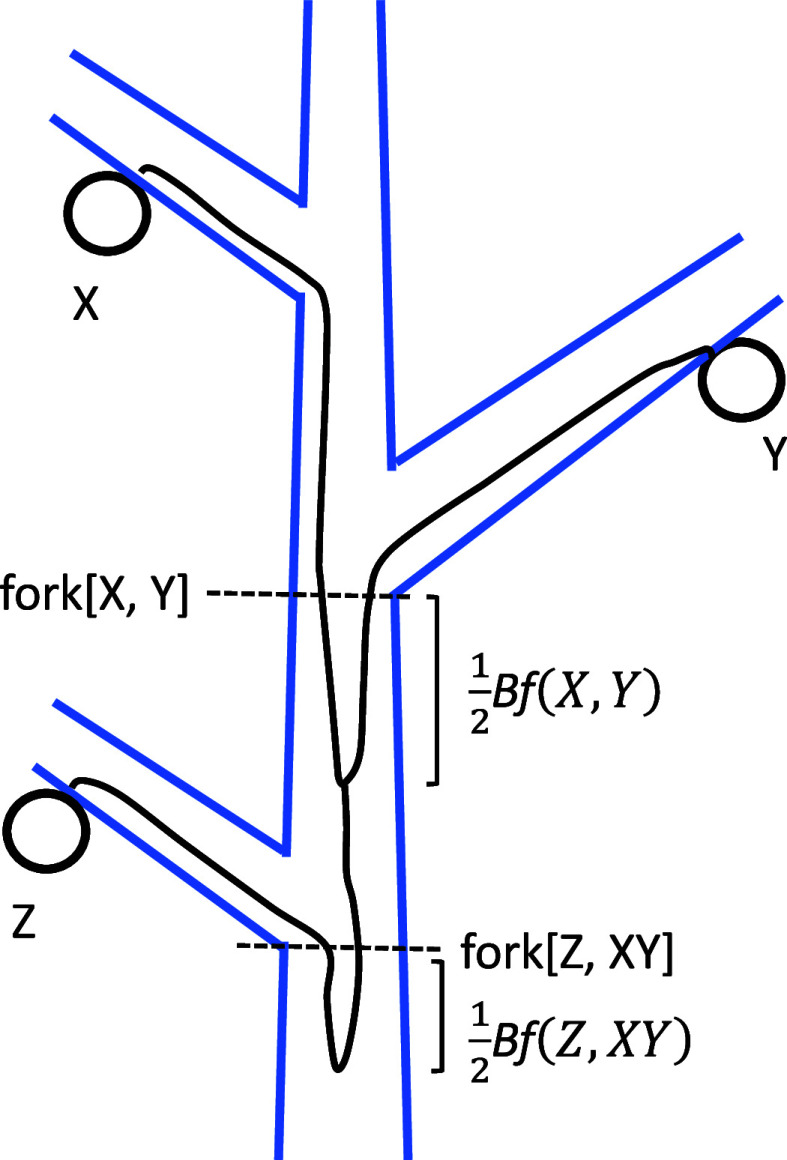

## 1. Introduction

Trees have long lifespans ranging from tens to hundreds of years, during which their trunks and branches continue to grow. Somatic mutations occur due to errors in genome replication during cell division and failures in DNA damage repair following physical or chemical disturbances. These mutations arise and accumulate in different branches, leading to genetic differentiation within an individual tree (Tomimoto & Satake, 2023). Thanks to recent developments in genomic analysis technologies such as next-generation sequencing, we have become able to identify genetic patterns caused by somatic mutations (Duan et al., 2022; Hanlon et al., 2019; Hofmeister et al., 2020; Orr et al., 2020; Perez-Roman et al., 2022; Plomion et al., 2018; Reusch et al., 2021; Schmid-Siegert et al., 2017; Schmitt et al., 2022; Wang et al., 2019; Zahrdadníková et al., 2020). The physical structure of the tree, with its branches, exhibited a topology similar to the molecular phylogenetic trees of cells sampled from different branches (Perez-Roman et al., 2022; Satake et al., 2023), suggesting that somatic mutations and epimutations accumulate as shoots elongate.

In plant tissues, somatic genetic variations can contribute to the next generation because reproductive organs (flowers and fruits) originate from stem cells in the shoot apical meristems (abbreviated as SAM). Gametes, including eggs and pollen, can undergo genetic diversification through somatic mutations, thereby contributing to genetic variation among offspring (Plomion et al., 2018; Sutherland & Watkins, 1986; Whitham & Slobodchikoff, 1981). This differs from animals with unitary structures, where germ lines differentiate from soma in early developmental stages.

In a previous article (Iwasa et al., 2023), we studied a mathematical model describing how the genetic pattern of a shoot is determined by the behavior of stem cells in the meristem. We evaluated the phylogenetic distance between cells sampled from different portions of a shoot, indicating their genetic difference due to somatic mutations accumulated during shoot elongation. Stem cells in the SAM normally undergo asymmetric cell division, producing successor stem cells and differentiated cells. However, occasionally, a stem cell may fail to leave its successor stem cell. Subsequently, to recover the stem cell number, the vacancy is filled by the duplication of one of the nearest neighbour stem cells. Because cell walls prevent stem cells from exchanging their positions, this leads to the genetic differentiation of cells according to the angle around the shoot and a larger genetic variance accumulated among cells in the body (Iwasa et al., 2023).

In this article, we aim to highlight the significance of somatic mutations (and epimutations) in generating genetic diversity among reproductive organs of the same individual and their evolutionary effects through modifying individual fitness and the population genetic variance. For analysis, we again adopt a cell lineage-based model (Chen et al., 2024).

We first note that there are several different aspects regarding the pattern of genetic variation of cells within a single individual tree. To clarify these differences, we introduce three indexes calculated based on phylogenetic distance, defined as the length of ancestral stem cell lineages between sampled cells (Iwasa et al., 2023). They depict different aspects of within-individual genetic diversity. Subsequently, we discuss how these indexes might contribute to various effects on evolutionary processes. Evaluating the evolutionary effects is closely related to population genetic theories developed for the evolution of sex, recombination, and mutation. In this article, the term ‘tree’ consistently refers to a large woody perennial plant typically having a single main stem or trunk. To indicate the evolutionary history and relationships of cells, we use the term ‘phylogeny.’

## 2. Background information

In this section, we summarize the basis for the discussion of the accumulation of somatic mutations and their patterns. First, we outline the characteristics of tree structures. Subsequently, we explain phylogenetic distance, defined as the length of ancestral phylogeny between two sampled cells until their common ancestral cell, which provides the basis for all the methods used in the remainder of the article.

### 2.1 Characteristics of tissue structure of trees

Here, we highlight characteristics of tree tissue structure, some of which are shared with modular animals (e.g., corals and sponges; Vasquez Kuntz et al., 2022).

**Figure 1.**
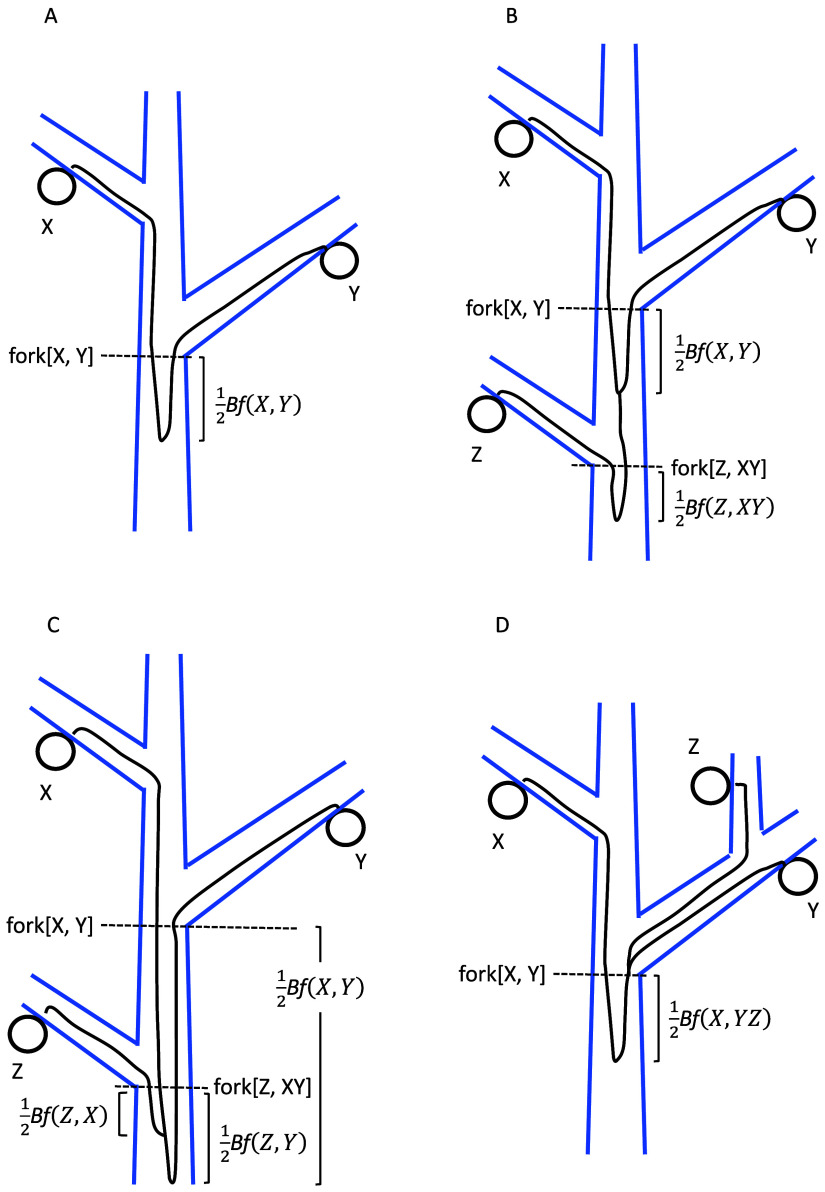
Scheme of ancestral cell lineages connecting sampled cells on different branches of a tree. Notation of symbols and explanations are provided in the main text and Part A of the SM. (A) X and Y are sampled cells. The ancestral cell lineages of X and Y converge at the same location, marked as fork[X, Y]. Their ancestral cell lineages then merge below it at a distance of 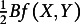 . (B) Three cells, X, Y and Z, are sampled from different branches. The coalescence of ancestral lineages of X and Y occurs below fork[X, Y] at a distance of 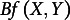 . It takes place above fork[Z, XY], which represents the forking between ancestral lineage of Z and the common ancestral cell lineage of X and Y. The coalescence of Z and XY occurs below fork[Z, XY] at a distance of 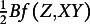 . (C) Three cells X, Y, Z are sampled. The coalescence of A and B does not occur above fork[Z, XY], where the shoot apocal meristem (SAM) includes three ancestral cells of X, Y and Z. (D) The coalescence of Y and Z does not occur within the lateral branch. Instead, it occurs at fork[X, YZ] as a result of bottleneck in the stem cell population in the SAM when the lateral branch is spliced.

- No clear distinction between germ line and soma:
  In plant tissues, somatic mutations accumulated during shoot elongation may contribute to reproductive organs and gametes (eggs and pollen), affecting the genetic content of offspring.
- Shoot apical meristems on different branches:
  In trees, each shoot (trunk or branch) contains a shoot apical meristem (SAM) with a small number of stem cells. The SAMs are physically separated and located at the tips of different branches. This structure facilitates independent accumulation of somatic mutations between branches, leading to their genetic differentiation.
- Before-forking portion of ancestral cell lineages:
  Stem cells undergo asymmetric cell division, creating both a successor stem cell and a differentiated cell. The differentiated cells then undergo a finite number of duplications, increasing in number and size to form a portion of a shoot. If the stem cells perform asymmetric cell division with a high probability, different stem cell lineages accumulate somatic mutations independently, leading to genetic heterogeneity of stem cells. The ancestral lineages of two different stem cells in the SAM have some distance between their common ancestor and the sampled location as illustrated in Figure 1. This is referred to as the ‘before-forking’ portion (Tomimoto et al., 2023). Its magnitude depends on the probability of failure in asymmetric cell division of stem cells (Iwasa et al., 2023).
- Circular genetic differentiation around a shoot:
  When a stem cell fails to leave its successor, the stem cell line is replaced by a copy of a neighbouring stem cell, as cell movement is constrained by the cell wall. This produces circular genetic differentiation of stem cells and leads to larger genetic diversity among them than when they are well mixed (Iwasa et al., 2023). Possible roles of the layer structure in the meristem have also been discussed (Klekowski et al., 1985; Pineda-Krch & Lehtilä, 2002).
- Distinction between the main and lateral branches:
  When a lateral branch is formed at a main branch, the stem cells of the axillary meristem of the lateral branch are sampled from the SAM of the main branch. In many trees, only a small fraction of stem cells in the main branch contribute to the axillary meristem (Tomimoto et al., 2023; Tomimoto & Satake, 2023). This results in an asymmetry between the two stem cell lineages sampled above the forking. As illustrated in Figure 1(D), the main branch maintains multiple ancestral cell lineages just below the forking, while cell lineages from the lateral branch coalesce at the forking. Main and lateral branches at a forking may be distinguished by comparing the genetic diversity among cells of the two branches.

### 2.2 Phylogenetic distance between cells

We consider the following situation: A shoot consists of cells derived from a small number of stem cells in the SAM located at the tip. A stem cell undergoes asymmetric cell division and produces one successor stem cell and one differentiated cell. The differentiated cells increase in number through a finite number of cell divisions, grow in cell size, form a portion of the shoot, and lift the SAM. This process continues and creates a shoot with the current SAM on the tip and the lower portion of the shoot reflecting an earlier genetic state of stem cells in the SAM (Iwasa et al., 2023).

All cells forming above-ground plant body are ultimately derived from stem cells in the SAM. As the shoot elongates, novel mutations accumulate. Hence, the genetic distance between two cells can be evaluated by the length of the ancestral lineage connecting them. Iwasa et al. (2023) defined phylogenetic distance 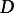 as the physical length along a branch, measured in units of cm or m. The genetic distance, representing the number of genomic differences between cells, is calculated by multiplying 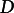 by the mutation rate per physical shoot length. Mutations occur due to errors in cell division and failures of DNA damage repair, with the rates proportional to the number of cell divisions and to the calendar time, respectively. We assume an infinite-site model where the same mutation does not occur in any stem cell, and no back mutation occurs. Comparing trees between fast and slow growing tropical species, Satake et al. (2023) concluded that the mutation rate proportional to the number of calendar years was much more important than the other type.

While Iwasa et al. (2023) discussed 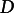 between two cells sampled from the same shoot, in this article, we consider the cases where the sampled cells are on different branches. Figure 1(A) illustrates an example of ancestral cell lineages connecting two sampled cells X and Y, located on different branches spliced from the main branch (trunk). The location labelled as fork[X, Y] represents the forking point between X and Y, where their ancestral cell lineages of cells X and Y converged to the same SAM. These two ancestral stem cells may or may not be identical, because a SAM contains multiple stem cells. If they are different, there is a distance between their common ancestor stem cell and fork[X, Y]. We call this portion as the ‘before forking’ segment (Tomimoto et al., 2023) and denote the length of the path between two ancestral stem cells at fork[X, Y] by 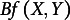 . The physical distance between the common ancestor cell and fork[X, Y] is half of 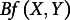 and was called ‘coalescent length’ between the two ancestral cells at fork[X, Y] (Iwasa et al., 2023)

The before-forking portion may extend to the base of the shoot. However, there is a possibility that their common ancestral stem cell exists in the middle of the shoot. This phenomenon, known as ‘coalescence’, can occur because a stem cell in the SAM sometimes fails to leave its successor stem cell. When such a failure occurs, another stem cell in the SAM duplicates to replenish the stem cell count. Consequently, a stem cell line is replaced by another, and the ancestral cell lineage of a focal stem cell shifts its location (Iwasa et al., 2023).

The phylogenetic distance between X and Y is defined as the length of their stem cell lineage since their common ancestor cell (Figure 1(A)), given by:(1)

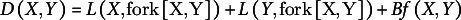

where the first term on the right-hand side represents the physical length along the branch between sampled cell X and the forking (see Iwasa et al., 2023 for definition of 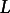 ). The second term represents a similar quantity for sampled cell Y. Detailed explanations are provided in Part A of the Supplemental Material (abbreviated as SM). The sum of the first and second terms on the right-hand side of Eq. (1) is the after-forking portion of the phylogenetic distance. The last term indicates the before-forking term, which increases with the genetic diversity of stem cells within the SAM.

Figure 1(B) illustrates the case of three sampled cells from different branches. The ancestral cell lineages of X and Y coalesce below fork[X, Y] by 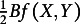 . Their common ancestral cell lineage then continues downward. The forking between their common ancestral lineage and the ancestral cell lineage of Z is denoted by fork[Z, XY]. In Figure 1(B), the common ancestor of X and Y is above fork[Z, XY]. Coalescence of all three ancestral lineages occurs where the ancestral lineage of Z and the common ancestral lineage of X and Y coalesce. It is located below fork[Z, XY] by length 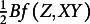 . For further details, refer to Part A of the SM.

Figure 1(C) illustrates the case ancestral cell lineages of X and Y do not coalesce above fork[Z, XY]. The ancestral cell lineages have three different ancestral cells at fork[Z, XY]. We observe a coalescent process of these three cells. In Figure 1(C), the ancestral cell lineages of X and Z coalesce first, followed by the coalescence of their common ancestral lineage with the ancestral lineage of Y.

Figure 1(D) illustrates the asymmetry between the main and lateral branches. The ancestral cell lineages of Y and Z do not coalesce within the lateral branch but they coalesce where the lateral branch is spliced because the stem cells of the SAM of the lateral branch were derived from a single stem cell in the SAM of the main shoot. In contrast, the main branch keeps the diversity, as illustrated by that the ancestral cell lineage of X was kept separate from the common ancestral cell lineage of Y and Z.

In Part B of the SM, we delve into the variance in the number of genetic differences between two sampled cells. It originates from the Poisson distribution with a mean proportional to the phylogenetic distance and also from the uncertainty of the ancestral cell lineage. Because this article aims to illustrate the various facets of within-individual genetic variation, our emphasis lies on the mean values of the indexes in the main text.

**Figure 2.**
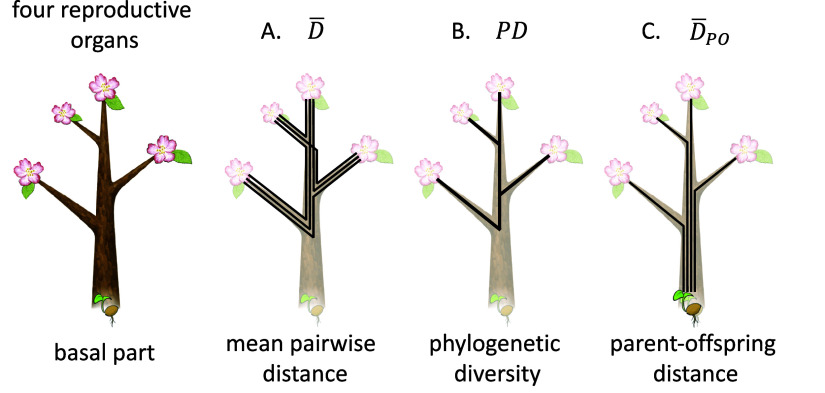
Three indexes for the genetic diversity of multiple reproductive organs of a single tree. We consider a tree with four reproductive organs on different branches (see the figure on the left). The three different indexes are: (A) mean pairwise phylogenetic distance 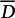 ; (B) Phylogenetic diversity 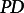 ; and (C) parent-offspring genetic distance 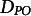 . Refer to the text for the explanations.

## 3. Three indexes

In this section, we will explain three indexes that characterize different aspects of within-individual genetic variation.

### *Mean pairwise phylogenetic distance between reproductive organs* 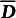


3.1

A tree may have many reproductive organs, producing seeds or flowers, with the latter producing pollen to fertilize the seeds of other trees. Let 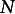 be the number of reproductive organs of a tree. A reproductive organ may be an inflorescence, which may later develop into multiple fruits. The size of a reproductive organ determines the number of seeds it produces, which in turn affects the number of offspring. We represent relative sizes of these organs as 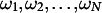 , which satisfy 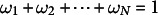 . The phylogenetic distance between organs 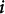 and 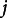 is denoted as 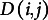 , which is defined by Eq. (1) (see Figure 2(A)).

Here, we define the mean pairwise phylogenetic distance among all the reproductive organs of an individual tree as follows:(2)

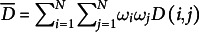

where 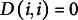 . This equals to the mean phylogenetic distance between two randomly chosen seeds. The genetic diversity is quantified by 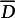 given in Eq. (2), multiplied by the mutation rate per unit shoot length.

Cells from the focal tree undergo meiosis, become haploid gametes and experience syngamy with the gametes produced by other trees. Hence, tree structure with a larger 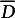 realizes genetically more diverse offspring.

### *Phylogenetic diversity of multiple stem cells of a tree* 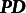


3.2

Phylogenetic diversity (abbrev. 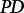 ) is defined as the sum of branch lengths of phylogeny (Figure 2(B)). It was first proposed by Faith (1992) as a measure of the importance of different species (or taxonomic units) in conservation biology, and has been adopted widely to evaluate the diversity of a group of multiple species (microbes; Sogin et al., 2006; Lauber et al., 2009). In the classical gene-genealogy theory, the sum of branch lengths of molecular phylogeny is calculated when those genomes are sampled from a single well mixed population. Watterson (1975) defined the expected number of segregating sites, denoted by 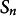 , as the product of mutation rate 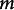 and the sum of branch lengths in gene phylogeny.

We apply 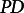 to quantify the genetic diversity of reproductive organs of a single individual tree, treating them as taxonomic units. Note that sampling was conducted in a population with geographic structures characterized by separation into multiple SAMs at different branches and a circular genetic structure within each SAM.

Both mean pairwise phylogenetic distance 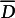 and phylogenetic diversity 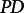 measure the genetic differences between reproductive organs and evaluate the variability among offspring (i.e., seeds and young trees). However, these two metrics emphasize different aspects of the genetic diversity among offspring. The phylogenetic diversity 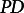 is proportional to the total number of haplotypes included in the whole phylogeny, giving equal weight to genotypes that produce numerous seeds and those that produce a single seed. In contrast, the mean phylogenetic distance between organs 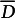 considers branches with many seeds more important than those with few sees.

**Figure 3.**
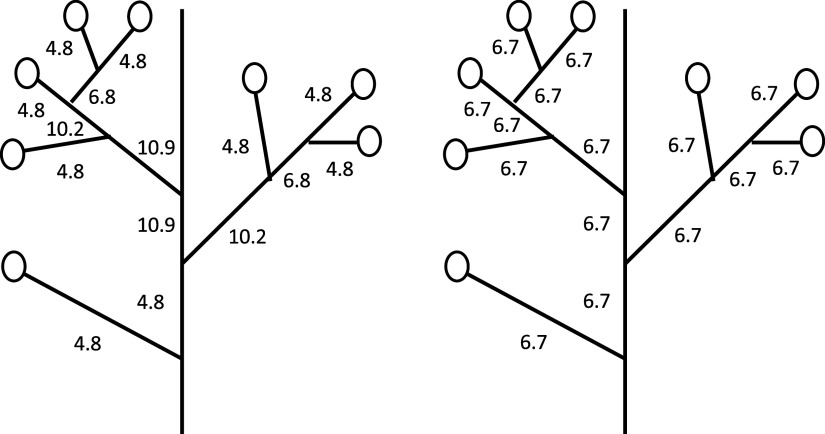
Difference in the relative importance of branches in a tree when calculating 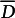 and 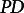 . Open circles indicate reproductive organs (flowers and fruits) on the branches of a tree. In this scenario, the same number of seeds is produced per reproductive organ, and their weights are equal: 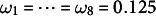 . (A) The relative importance of branches in calculating 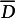 . They are normalized to ensure their sum equals 100. In this case, the importance of a branch is proportional to the number of ancestral cell lineages connecting two reproductive organs. (B) The relative importance of branches in calculating 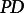 .

Additionally, even when all the reproductive organs have equal weight ( 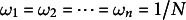 ), 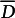 and 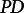 assign different weights to various branches. Figure 3 illustrates a tree with eight reproductive organs ( 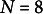 ). In Figure 3(A), each branch has an importance proportional to the number of ancestral lineages connected two organs. Note that removing a branch results in two disconnected trees. If the number of organs included in these trees are 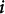 and 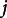 , the weight for the branch is proportional to 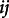 . In Figure 3(A), we indicated the importance of each branch in calculating 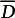 , with the values normalized to ensure their sum equals 100. In contrast, Figure 3(B) illustrates the equal importance of each branch for 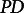 , with the same value assigned to all branches. Comparing these two, we can conclude that 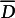 assigns more weight to branches in the central portion than those in the marginal portion of the tree.

#### 3.2.1 Phylogenetic diversity of stem cells sampled at the same position

Figure 1(C) illustrates the case where the ancestral cells of three sampled cells, X, Y and Z, are different cells in the same SAM. We need to evaluate the sum of branch lengths of ancestral lineages of these three cells until the coalescence. In this section, we consider the phylogenetic diversity of more than two stem cells in the same SAM along the shoot.

Let 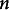 be the number of stem cells in the SAM. They are arranged in a circular manner, with the nearest neighbours separated by an angle of 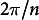 radians. The number of sampled stem cells is 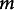 , and they are separated from their neighbors by 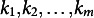 ( 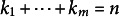 ). If a stem cell fails to leave its successor stem cell, the location is filled by a copy of its nearest neighbor. This occurs at a rate 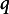 per unit shoot length. Then the location of the ancestral lineage of a sampled cell shifts by one, either to the left or right. The mean phylogenic diversity of sampled stem cells, denoted by 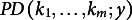 , depends on 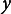 , the distance of the sampled location from the base of the shoot.

**Figure 4.**
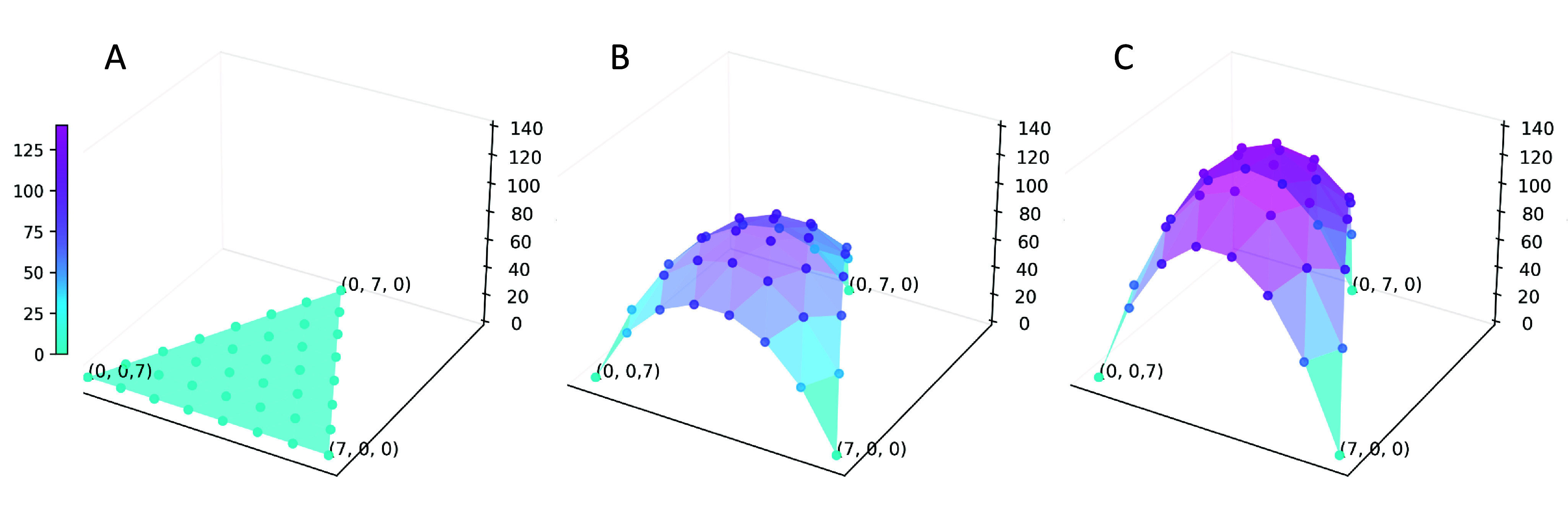
Phylogenetic diversity of three stem cells sampled from the same SAM. The intervals between sampled stem cells are denoted as: 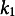 , 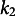 , and 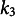 , which are positive integers satisfying 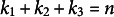 . Two horizontal axes represent 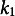 and 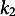 . The calculation is made based on the system of differential equations given in Eq. (B.2) in Part C of the SM, showing 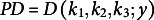 . Three parts correspond to different distances from the base of the shoot: (A) 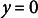 ; (B) 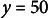 ; (C) 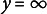 . Parameters are: 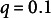 , 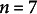 , and 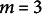 .

In Part C of the SM, we derive a differential equation for 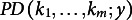 and obtain the solution considering the boundary conditions. Figure 4 illustrates how 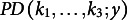 increases with 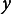 . In the limit of very large 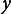 ( 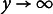 ), we have the following solution:(3)

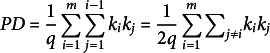

which indicates the total sum of the coalescent lengths of 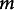 stem cells if the sampled location is far from the base of the shoot. 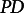 is large when stem cells have a high probability of leaving their successor cells ( 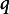 is small). It also depends on the intervals between the sampled stem cells 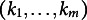 . We can rewrite Eq. (3) as follows:(4)

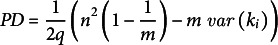

where 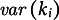 is the variance of the length of intervals: Phylogenetic diversity reaches its maximum when the sampled cells are equally spaced. When the stem cells are sampled with equal intervals ( 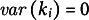 ), 
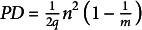
 holds.

When we sample just two cells ( 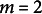 ), 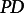 is the same as the phylogenetic distance 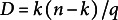 , which was the basis of index 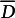 calculated in the last section.

### *Parent-offspring distances* 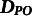


3.3

The genetic diversity generated by somatic mutations per generation contributes to the genetic variation of the whole population, which controls the speed of adaptation of traits under anthropogenetic environmental changes. The genetic variance of a quantitative trait in the entire population increases by mutations and decreases due to random genetic drift and stabilizing selection (Lande, 1976, 1981). The rate at which genetic variance is produced per generation is calculated based on the trait change from the parent to offspring, rather than the difference between offspring. Hence, to evaluate the contribution to the genetic variation of the population, we need to know the phylogenetic distance between parent and offspring (Figure 2(C)). However, both 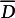 and 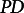 only measure the genetic variation among offspring.

We here consider the parent-offspring phylogenetic distance as follows:(5)

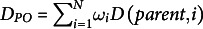

where 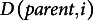 is the phylogenetic distance between the parent genome and the *i*th reproductive organ: 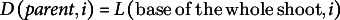 , which is simply equal to the physical length between the base of the whole shoot and the reproductive organs. 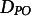 is independent of the genetic diversity of stem cells in the SAM, unlike the two other indexes.

These three indexes capture different aspects of within-individual genetic variation. This can be illustrated by examining a simple case. In Part D of the SM, we analysed the three indexes in a model tree with branching architecture studied in Tomimoto et al. (2023), which exhibit different dependence on the number of stem cells in the SAM.

## 4. Discussion: evolutionary impacts

Trees exhibit several characteristic aspects of tissue structure that differ from many animals (unitary organisms). As a result, trees accumulate somatic mutations efficiently, which contribute to the genetic variability of offspring. The selective advantage of somatic mutations expressed in the current generation have been discussed: For example, somatic mutations can lead to diversification in defence chemicals between branches, reducing the harm of herbivores and pathogens (Antolin & Strobeck, 1985; Folse & Roughgarden, 2012; Gill et al., 1995). In addition, somatic mutations can modify the genome of reproductive organs and the genomic composition of gametes (eggs and pollens), resulting in genetically diverse offspring (Schoen & Schultz, 2019; Whitham & Slobodchikoff, 1981). These may have diverse evolutionary effects. In this section, we will address these effects and discuss how three indexes for the genetic diversity of somatic mutations assess different evolutionary effects.

### 4.1. Generating genetic variations

We may ask whether we can justify the argument that the tissue structure of trees might have evolved as a result of promoting a high rate of mutations in the next generation.

In Part E of the SM, we briefly summarize the conclusions of population genetics theory for the evolution of mutation rates. In the simplest populations (constant environments, no spatial structure), the mutation rate controlled by neutral modifiers evolves towards lower values (Altenberg et al., 2017; Karlin, 1975; Karlin & McGregor, 1974; Liberman et al., 2011). In populations with various structures, a higher mutation rate can evolve. The key elements of these processes were originally studied in the context of the evolution of sex and recombination. Among them, processes such as environmental fluctuations (Ishii et al., 1989; Sasaki & Iwasa, 1987), interactions with antagonistic species (Haraguchi & Sasaki, 1996, 1997; Sasaki & Haraguchi, 2000), spatial heterogeneity, and sibling competition (Bell, 1982; Douge & Iwasa, 2017a, 2017b; Maynard Smith, 1978; Williams, 1975) have been shown to potentially favour the evolution of a high mutation rate.

Many of these theoretical studies discuss the evolution of the rate of switches between functional alleles that can confer advantages in different environments. However, in most situations, mutations arise due to errors in replication or repair, and they often result in producing many defective and malfunctional genes. It is very difficult for a high mutation rate to be advantageous in general circumstances, except for special mechanisms appearing in host–pathogen interactions (Metzgar & Willis, 2000; Rosenberg et al., 1998). The observed positive rate of mutation in genomes is considered to have evolved not because of the advantage of higher mutation rate but determined by the high cost of reducing the mutation rate further by investing in more accurate repair or duplication of the genome (Lynch, 2010, 2011).

Therefore, we can conclude that the tissue structure of trees has evolved not because of a high mutation rate but because of other reasons, such as photosynthetic efficiency, competition with neighbouring individuals, defence against pathogens or predators, mechanical strength, etc. Mutations produced by trees that contribute to the next generation are considered as ‘byproducts’ of the tissue structures.

Even if the tissue structure of trees evolved primarily for other reasons, examining the evolutionary effects quantitatively is important. Among many processes that may provide a fitness advantage for having a higher mutation rate, the two most promising ideas are (1) the antagonistic genotype-specific interaction with pathogens and herbivores, and (2) situations characterized by significant spatio-temporal variation and intense sib-competition (refer to Part E of the SM). These promote the advantage of producing a few diverse offspring, rather than having many similar offspring. It is worthy to quantify these fitness effects in the field. They are likely to be important for genes involved in antagonistic interactions with pathogens and herbivorous insects, especially in species with strong spatial variation and intense sib competition. These are plausible for trees with gravity-dispersed seeds (barochory). Additionally, separate from the fitness benefits to the parent tree, there exists (3) an effect of increasing the genetic variance of the whole population, which governs the population’s evolutionary capacity to respond to environmental changes.

### 4.2. Efficient elimination of deleterious mutations

An analysis of the next-generation sequencing data in trees concluded that somatic mutations behave as neutral variations during branch elongation but suffer deleterious effects when they form the next generations (Ally et al., 2010; Satake et al., 2023), which was different from the expectation of the population genetic theory (Otto & Orive, 1995). This implies that somatic mutations are maintained without receiving strong selection within an individual tree, suggesting a novel theoretical idea for the evolutionary advantage of tree tissue structure: Trees might be able to effectively eliminate harmful deleterious genes, provided that the deleterious mutations work synergistically.

Mutation accumulation experiments suggested that the fitness of a genome including 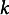 deleterious genes is represented as 
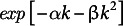
 with 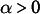 and 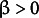 (Mukai, 1969), implying that the effect of a few mutations is small, but with sufficiently many mutations, fitness declines rapidly in an accelerating manner (Maynard Smith, 1978). This type of epistasis eliminates deleterious mutations without much reduction in the population’s mean fitness, as emphasized in the theory of the evolution of sex (Hurst & Peck, 1996; Kondrashov, 1988, 1993; Kondrashov & Crow, 1988).

We can compare a tree that accumulate somatic mutations over hundreds of years with a large genet of a clonal plant that extends through many small individuals (ramets). In the clonal plant, deleterious mutations are expressed each time a ramet is formed, as shown in seagrass (Yu et al., 2020). In contrast, in tree, the mutations accumulate without being exposed to selection and receive strong selection only during the reproductive stages. Different branches of a tree may accumulate different numbers of deleterious mutations. If the mutations interact synergistically during reproduction, a small fraction of seeds may carry many deleterious mutations, leading to significantly reduced fitness, while others may not exhibit as much deleteriousness. This, coupled with strong sib competition, could efficiently remove deleterious mutations and improve the reproductive success of the parent tree compared with the corresponding clonal plant.

At this moment, empirical evidence for the extent of synergism was not decisive: Synergism was supported in experiments with *Drosophila* (Mukai, 1969) but not detected in a study with *E. coli* (Elena & Lensky, 1997). Empirical studies on the accumulation of somatic mutations in trees and clonal plants yielded varying conclusions: Ally et al. (2010) reported a decline in fertility, Bobiwash et al. (2013) estimated the accumulation of deleterious mutations in a long-lived clonal shrub. In contrast, Cruzan et al. (2022) found within-individual selection in perennial herbs resulting in the production of advantageous mutations in some shoots, while Alejano et al. (2019) observed no correlation between the fitness of offspring and the age of the parent tree. More empirical studies are needed.

### 4.3 Indexes measuring different aspects of novel genetic variation

In this article, we discussed three indexes for the genetic diversity of somatic mutations within an individual tree. They quantify the amount of genetic diversity in different aspects.

The additive genetic variance of a population is crucial in determining its response to environmental changes. Somatic mutations in trees may increase the genetic variance of the population. To assess this effect, 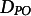 is important as it represents the magnitude of parent-offspring distance produced per generation.

In contrast, to measure the contribution to the fitness of the parental tree, we need to count the number of surviving offspring relative to other trees in the same population. Both 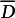 and 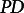 measure the genetic diversity of offspring produced by each individual tree, rather than their effect on the entire population.

If we compare the two indexes, 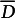 and 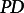 , the phylogenetic distance 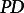 is proportional to the total number of haplotypes included in the whole phylogeny. It assigns equal importance to genotypes with different numbers of seeds. In contrast, the mean phylogenetic distance between organs 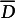 considers a branch with many seeds more important than another with only a few seeds.

In situations where the majority of seeds can survive and engage in intense sibling competition, the number of genetically distinct offspring becomes crucial, rendering multiple copies of the same genotype irrelevant. In such cases, 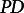 (phylogenetic diversity) holds more significance than 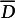 (mean pairwise distance). However, if a large fraction of offspring die early in their life before they start to compete with each other, a genotype existing as a single copy is likely to be eliminated stochastically. In such cases, producing many offspring of the same genotype makes sense to realize at least one individual can join in the sib competition. In such scenarios, 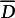 , which considers the relative number of seeds, may be more appropriate than 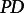 . For further argument, refer to Part F of the SM.

In a species-rich environment such as tropical rain forests, the specialist herbivores and pathogens are abundant near the parent tree (Janzen, 1970). Having the offspring with traits different from the parent might be more important than having offspring that differ among themselves. In such a case, 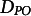 might be the most suited in evaluating the fitness contribution to the parent.

In short, the choice of the most suitable index for quantifying the genetic variation caused by somatic mutations should depend on which aspect of the many evolutionary effects one intends to evaluate.

To enhance our understanding of the role of tree tissue structure in accumulating somatic mutations, we require both theoretical studies to explore the evolutionary impacts of somatic mutations accumulated in trees and empirical research in the field that takes into account the success of all life stages, genetic structure, as well as both temporal and spatial variations.

## Supporting information

Iwasa et al. supplementary material Iwasa et al. supplementary material

## Data Availability

The authors declare that the data supporting the findings of this study are available within the paper, and its supplementary information files. Because the research is entirely theoretical, no empirical data are available.

## Supplementary material

The supplementary material for this article can be found at http://doi.org/10.1017/qpb.2024.9.
