# Supplementary material for: Genetic diversity within a tree and alternative indexes for different evolutionary effects
Source: Quant Plant Biol. 2024 Dec 6;5:e11. doi: 10.1017/qpb.2024.9 (PMC11706689; doi:10.1017/qpb.2024.9)
Supplement: Iwasa et al. supplementary material [file S2632882824000092sup001.pdf]

File S1: Supplemental Material

for

Genetic diversity within a tree and alternative indexes for different evolutionary effects

by

Yoh Iwasa, Sou Tomimoto, and Akiko Satake

Part A

Phylogenic diversity of cells within a single tree

Here, we explain how to evaluate the phylogenetic distance between two sampled cells and also how to assess the phylogenetic diversity among three or more sampled cells.

A.1 Phylogenetic distance

We denote the physical length along branch between the sampled cell X and the forking point fork[X, Y] as $L(\mathrm{X}, \mathrm{fork}[\mathrm{X}, \mathrm{Y}])$. Similarly we denote the length between cell Y and fork[X,Y] as $L(\mathrm{Y}, \mathrm{fork}[\mathrm{X}, \mathrm{Y}])$. The length of the path connecting X and Y along branches "after forking" is defined as the sum:

$$Af(X, Y) = L(X, \mathrm{fork}[\mathrm{X}, \mathrm{Y}]) + L(Y, \mathrm{fork}[\mathrm{X}, \mathrm{Y}]) \tag{A.1a}$$

The phylogenetic distance between two sampled cells, X and Y, is

$$D(X, Y) = Af(X, Y) + Bf(X, Y) \tag{A.1b}$$

In a similar manner, we define the after-forking part of the distance between X and Z:

$$Af(X, Z) = L(\mathrm{X}, \mathrm{fork}[\mathrm{XY}, \mathrm{Z}]) + L(\mathrm{Z}, \mathrm{fork}[\mathrm{XY}, \mathrm{Z}]) \tag{A.2a}$$

Note that fork[XY, Z] indicates that the location along shoot where the ancestral

lineage of Z intersects with the common ancestral lineage of X and Y (See Fig. A1). The phylogenetic distance between X and Z is as follows:

$$D(X,Z) = Af(X,Z) + Bf(XY,Z) \tag{A.2b}$$

We can define the distance between Y and Z in a similar manner.

$$Af(Y,Z) = L(Y,\text{fork[XY, Z]}) + L(Z,\text{fork[XY, Z]}) \tag{A.3a}$$

$$D(Y,Z) = Af(Y,Z) + Bf(XY,Z) \tag{A.3b}$$

The before-forking portion is twice as long as the coalescent length discussed in Iwasa et al. (2023). It depends on the angle difference between two stem cells, indicating by k $(k = 0, 1, 2, \ldots, n)$. This corresponds to a radian angle of $2\pi k/n$. It is challenging to determine the angle of the ancestral cells of a sampled cell located on a branch. Additionally, due to the failure of a stem cell to leave its successor, an ancestral stem cell lineage can occasionality be superseded by another stem cell line, causing a movement of the ancestral cell lineage (Iwasa et al. 2023). As a reesult, we randomize the angle difference and then consider the mean value of before-forking portion:

$$\overline{Bf} = \frac{1}{n}\sum_{k=1}^{n}\frac{k(n-k)}{q} = \frac{n^2-1}{6q} \tag{A.4}$$

All the other measurements represent the physical distance along branches.

A.2 Phylogenetic diversity PD

The phylogenetic diversity of the sampled cells X, Y, and Z becomes as follows:

The undirected graph connecting X, Y, and Z by ancestral lineages is determined by summing the lengths of edges between the coalescent point between X

and Y and the three sampled cells:

$$PD[X,Y,Z] = \left\{L(\mathrm{X},\mathrm{fork}[\mathrm{X},\mathrm{Y}]) + \frac{1}{2}Bf(X,Y)\right\} + \left\{L(\mathrm{Y},\mathrm{fork}[\mathrm{X},\mathrm{Y}]) + \frac{1}{2}Bf(X,Y)\right\}$$

$$+\left\{L(\mathrm{fork}[\mathrm{X},\mathrm{Y}],\mathrm{fork}[\mathrm{XY},\mathrm{Z}]) - \frac{1}{2}Bf(X,Y) + Bf(XY,Z) + L(\mathrm{Z},\mathrm{fork}[\mathrm{XY},\mathrm{Z}])\right\}$$

$$= \{L(\mathrm{X},\mathrm{fork}[\mathrm{X},\mathrm{Y}]) + L(\mathrm{Y},\mathrm{fork}[\mathrm{X},\mathrm{Y}]) + L(\mathrm{Z},\mathrm{fork}[\mathrm{XY},\mathrm{Z}])\}$$

$$+L(\mathrm{fork}[\mathrm{X},\mathrm{Y}],\mathrm{fork}[\mathrm{XY},\mathrm{Z}]) + \left\{\frac{1}{2}Bf(X,Y) + Bf(XY,Z)\right\}$$

The last expression indicates that PD is the sum of terms calculated from the physical length along branches and the terms related to the before-forking portion. The former can be measured simply from the physical length, while the latter is connected tp the genetic diversity of stem cells within the SAM. This genetic diversity depends on the number of stem cells in the SAM, as well as the frequency of failure in asymmetric cell division and which stem cells supersede others when a stem cell fails to leave its successor stem cell (Iwasa et al. 2023).

Part B

Variance of the number of genetic differences between cells and phylogenetic distance.

If we can identify the ancestral lineage of two sampled cells, their phylogenetic distance is equal to it. The number of genetic differences between i and j is a stochastic variable following Poisson distribution with mean proportional to their phylogenetic distance. It has some variance equal to the mean.

In general, we may not be able to identify the ancestral lineage between the two sampled cells. We have to consider additional variance caused by the uncertainty of the ancestral linage. Suppose that i and j are two different stem cells sampled from the same shoot apical meristem (SAM). The phylogenetic distance includes variance originated from the uncertainty of the coalescence location. The distance between the location of their coalescence and the forking follows an exponential distribution with the mean inversely proportional to the rate of events that a stem cell fails in leaving its successor, and the location is taken over by their neighboring stem cell lineage (refer to Iwasa et al., 2023). When we consider the genetic difference between i and j, we need to consider the Poisson distributed stochasticity as well as the uncertainty of the phylogenetic distance.

If we sample two cells located on different branches, we can identify the location of their forking point (Fork[i,j]). The phylogenetic distance between i and j is

$$D(i,j) = D(i, Fork[i,j]) + D(j, Fork[i,j]) + Bf(i',j') \quad \text{(B.1)}$$

where i' and j' are the stem cells at the fork$[i,j]$, i' is an ancestoral cell of i, and j' is an ancestoral cell of j, We need to note that i' and j' may be different stem cells

or they may be the identical stem cell. We may consider theses cases with probability $\frac{n-1}{n}$ and $\frac{1}{n}$, respectively, if we do not know the ancestral lineage of i and j. In the latter case the last term in Eq. (A.1) is set 0. Then $Bf(i', j')$ when $i' \neq j'$, has stochasticity as explained in the last paragraph (Iwasa et al. 2023).

As illustrated by this simple situation, we have uncertainty concerning the ancestral cell lineage between given sampled cells. In addition, concerning the number of genetic differences, there is a stochasticity caused by the Poisson process of stochasticity.

All the indexes discussed in this paper are calculated from the phylogenetic distances. In the present paper, we aim to clarify the differences between various aspects of genetic diversity by focusing on the mean values of phylogenetic distances.

Part C

Derivation of the phylogenetic diversity (PD) of multiple sampled stem cells within the same SAM.

Suppose we have sampled three cells at locations: l_0, l_1, and l_2, which satisfy $0 \le l_0 \le l_1 \le l_2 \le n$. The intervals between these cells are denoted as k_1, k_2, k_3, which are defined as follows: $k_1 = l_1 - l_0$, $k_2 = l_2 - l_1$, and $k_3 = n + l_0 - l_2$. These intervals satisfy the following conditions:

$k_1 \ge 0,\ k_2 \ge 0,\ k_3 \ge 0,\ k_1 + k_2 + k_3 = n.$

When one stem cell moves to the right by one, the interval in the left of the cell increases by one, which the interval in the right of the cell decreases by one. For example, when the ith cell moves to the right, l_i increases by one, k_i increases by one, and k_{i+1} decreases by one, simultaneously. If a cell moves to the left, the corresponding l_i decreases by one, k_i decreases by one, and k_{i+1} increases by one. The lengths of other intervals remain unchanged.

We first consider the case in which there are three cells, and k_1 and k_2 are integers within a triangular region on a plane: $k_1 > 0$, $k_2 > 0$, and $k_1 + k_2 < n$.

We adopt a continuous-time Markov chain, where only a single change occurs within a short interval. Let $D(k_1, k_2, k_3; y)$ represent the mean value of phylogenic diversity for three stem cells with angular intervals, k_1, k_2, k_3 around the shoot axis, sampled at location y from the base of the shoot. Please note that, for the sake of brevity, we us the notation D in this appendix, which refer to PD. Then we derive the following recursive formula:

$D(k_1, k_2, k_3; y + \Delta y) = 3\Delta y + (1 - 3q\Delta y)D(k_1, k_2, k_3; y + \Delta y)$

$$+\frac{q\Delta y}{2}[D(k_1+1,k_2-1,k_3;y)+D(k_1-1,k_2+1,k_3;y)$$

$$+D(k_1,k_2+1,k_3-1;y)+D(k_1,k_2-1,k_3+1;y)$$

$$+D(k_1-1,k_2,k_3+1;y)+D(k_1+1,k_2,k_3-1;y)] \quad \text{(C.1)}$$

As Δy approaches zero, we obtain

$$\frac{dD}{dy}=3+\frac{q}{2}\big(D(k_1+1,k_2-1,k_3)+D(k_1-1,k_2+1,k_3)-2D(k_1,k_2,k_3)\big)$$

$$+\frac{q}{2}\big(D(k_1,k_2+1,k_3-1)+D(k_1,k_2-1,k_3+1)-2D(k_1,k_2,k_3)\big)$$

$$+\frac{q}{2}\big(D(k_1-1,k_2,k_3+1)+D(k_1+1,k_2,k_3-1)-2D(k_1,k_2,k_3)\big) \quad \text{(C.2)}$$

where (k_1,k_2,k_3) represents all the points within the triangle. To save space, we do not display the dependence on y, although they are all functions that increase with y. In the limit of very large y, we have

$$0=3+\frac{q}{2}\big(D(k_1+1,k_2-1,k_3)+D(k_1-1,k_2+1,k_3)-2D(k_1,k_2,k_3)\big)$$

$$+\frac{q}{2}\big(D(k_1,k_2+1,k_3-1)+D(k_1,k_2-1,k_3+1)-2D(k_1,k_2,k_3)\big)$$

$$+\frac{q}{2}\big(D(k_1-1,k_2,k_3+1)+D(k_1+1,k_2,k_3-1)-2D(k_1,k_2,k_3)\big) \quad \text{(C.3)}$$

Here we introduce three operators, which modify a function $f(k_1,k_2,k_3)$. We define them as follows:

$$O^{12}[f(k_1,k_2,k_3)]=f(k_1+1,k_2-1,k_3)+f(k_1-1,k_2+1,k_3)-2f(k_1,k_2,k_3)$$

$$O^{23}[f(k_1,k_2,k_3)]=f(k_1,k_2+1,k_3-1)+f(k_1,k_2-1,k_3+!)-2f(k_1,k_2,k_3)$$

$$O^{31}[f(k_1,k_2,k_3)]=f(k_1-1,k_2,k_3+1)+f(k_1+1,k_2,k_3-1)-2f(k_1,k_2,k_3)$$

(C.4)

With these symbols, Eq. (C.3) is rewritten as follows:

$$0=3+\frac{q}{2}(O^{12}[L]+O^{23}[L]+O^{31}[L]) \quad \text{(C.5)}$$

When we apply this to the function $f(k_1,k_2,k_3)=k_1k_2$, we have the following results:

$O^{12}[k_1k_2] = (k_1+1)(k_2-1) + (k_1-1)(k_2+1) - 2k_1k_2 = -2$

$O^{12}[k_2k_3] = (k_2+1)k_3 + (k_2-1)k_3 - 2k_1k_2 = 0$

$O^{12}[k_3k_1] = 0$ (C.6a)

In a similar manner, we have

$O^{23}[k_1k_2] = 0,\ O^{23}[k_2k_3] = -2,\ O^{23}[k_3k_1] = 0$ (C.6b)

$O^{31}[k_1k_2] = 0,\ O^{31}[k_2k_3] = 0,\ O^{31}[k_3k_1] = -2$ (C.6c)

If we set $L(k_1,k_2,k_3) = C(k_1k_2 + k_2k_3 + k_3k_1)$, the following results holds:

$O^{12}[L] + O^{23}[L] + O^{31}[L] = -6C$ (C.7)

From Eq. (C.5), we have

$$0 = 3 + \frac{q}{2}(-6C)$$

This implies that Eq. (C.3) holds if $C = \frac{1}{q}$. Therefore, one candidate solution for Eq. (C.3) is as follows:

$D(k_1,k_2,k_3) = \frac{1}{q}(k_1k_2 + k_2k_3 + k_3k_1)$ (C.8)

Hence, Eq. (C.8) satisfies Eq. (C.3). However, to be considered a valid solution, it must also meet the boundary condition. The boundary condition relates to the consistency of the solution when one of k_1, k_2, k_3 is set to zero. In such a case, the coalescence of two of the three stem cells occurs, resulting in a model with two stem cells. This situation aligns with the model studied in Iwasa et al. (2023). Hence, we know that

$D = \frac{1}{q}k_1(n-k_1) = \frac{1}{q}k_1k_2$, if $k_3 = 0$. (C.9a)

$D = \frac{1}{q}k_2k_3$, if $k_1 = 0$. (C.9b)

$D = \frac{1}{q} k_3 k_1$, if $k_2 = 0$. (C.9c)

These conditions apply to the three edges of the triangular defined by Eq. (C.8). Consequently, we can conclude that $D(k_1, k_2, k_3)$ as presented in Eq. (C.8) is indeed the solution to (C.3). In other words, it is the solution to the differential equation (C.2) in the limit of as y approaches infinity.

Consider the scenario where a single coalescent event occurs. In this case, the operator of the differential equation is different from that of the three-stem cell case, as only two stem cells remain instead of three. In such a situation, the state point should lie on one of the three edges, rather than inside the triangle. Suppose it is the edge of $k_3 = 0$. On this edge, the following equation must hold:

$$\frac{dD}{dy} = 2 + \frac{2q}{2}\left(D(k_1 + 1, k_2 - 1) + D(k_1 - 1, k_2 + 1) - 2d(k_1, k_2)\right) \quad \text{(C.10)}$$

Note that the first term on the right-hand side is 2 instead of 3. This is because when one stem cell is replaced, both distances change, but we count the change in the distance twice because there are two stem cells involved. Both k_1 and k_2 must change if stem cells located at l_0 or l_1 are replaced. When l_1 moves to the right by one step, k_1 increases, and k_0 decreases by one. If l_1 moves to the left, the opposite changes occur. Additionally, the shift in l_0 occurs at the same rate with similar effects, and hence we must multiply by a factor of 2. Therefore, we need to obtain the solution of the following:

$$0 = 2 + \frac{2q}{2} O^{12} L(k_1, k_2) \quad \text{(C.11)}$$

The solution of this equation is $D = \frac{1}{q} k_1 k_2$.

If we consider four stem cells, the distance between adjacent stem cells are k_1, k_2, k_3, and k_4. The differential equation is as follows:

$$\frac{dD}{dy} = 4 + \frac{q}{2}\big(D(k_1+1, k_2-1, k_3, k_4) + D(k_1-1, k_2+1, k_3, k_4) - 2D(k_1, k_2, k_3, k_4)\big)$$
$$+\frac{q}{2}\big(D(k_1, k_2+1, k_3-1, k_4) + D(k_1, k_2-1, k_3+1, k_4) - 2D(k_1, k_2, k_3, k_4)\big)$$
$$+\frac{q}{2}\big(D(k_1, k_2, k_3+1, k_4-1) + D(k_1, k_2, k_3-1, k_4+1) - 2D(k_1, k_2, k_3, k_4)\big)$$
$$+\frac{q}{2}\big(D(k_1-1, k_2, k_3, k_4+1) + D(k_1+1, k_2, k_3, k_4-1) - 2D(k_1, k_2, k_3, k_4)\big) \quad \text{(C.12)}$$

Building on this, we define operators in a manner similar to what was explained earlier, resulting in the following differential equation:

$$\frac{dL}{dy} = 4 + \frac{q}{2}(D^{12}[L] + D^{23}[L] + D^{34}[L] + D^{41}[L]) \quad \text{(C.13)}$$

From this, the following represents a candidate solution for Eq. (C.13).

$$D = \frac{1}{q}(k_1k_2 + k_2k_3 + k_3k_4 + k_4k_1). \quad \text{(C.14)}$$

However, Eq. (C.14) does not satisfy the boundary condition on the plane $k_4 = 0$. With $k_4 = 0$, Eq. (C.14) yields $D = \frac{1}{q}(k_1k_2 + k_2k_3)$, which is incorrect. The correct solution on the plane $k_4 = 0$ is as follows:

$$D = \frac{1}{q}(k_1k_2 + k_2k_3 + k_3k_1). \quad \text{(C.15)}$$

To obtain Eq. (C.15) on plane $k_4 = 0$, we consider the following solution, which includes an additional term:

$$D = \frac{1}{q}(k_1k_2 + k_2k_3 + k_3k_4 + k_4k_1) + \frac{1}{q}k_3k_1 \quad \text{(C.16)}$$

This additional term has no effect because it vanishes:

$$(O^{12}[k_3k_1] + O^{23}[k_3k_1] + O^{34}[k_3k_1] + O^{41}[k_3k_1]) = 0 \quad \text{(C.17)}$$

Therefore, D as defined by Eq. (C.16) is the same as Eq. (C.13) within the tetrahedron region.

Similarly, for consistency on planes $k_1 = 0$, $k_2 = 0$, and $k_3 = 0$, we must introduce two additional terms:

$$D = \frac{1}{q}(k_1k_2 + k_2k_3 + k_3k_4 + k_4k_1) + \frac{1}{q}(k_1k_3 + k_2k_4) \quad \text{(C.18)}$$

This aligns with Eq. (C.13) within the tetrahedron and is consistent with the boundary condition on the planes.

Based on these results, we propose that the following solution for the general case of m, the number of stem cells:

$$D = \frac{1}{q}\sum_{i=1}^{n}\sum_{j=1}^{i-1} k_ik_j \quad \text{(C.19)}$$

Note that the sum is calculated only once for each pair of stem cells. Because

$$0 = m + \frac{q}{2}(O^{12}[D] + O^{23}[D] + .. + O^{m-1,m}[D] + O^{m1}[D]) = m + \frac{q}{2}\frac{1}{q}m(-2) = 0$$

(C.20)

Therefore, Eq. (C.19) satisfies the differential equation.

The boundary condition is derived from the fact that when one of the intervals becomes zero, say $k_i = 0$, the system decreases in dimension by one. However, the operator must be appropriately adjusted.

Let us consider the following operator.

$$O^{12}[D] + O^{23}[D] + .. + O^{k-1,k}[D] + O^{k,k+1}[D] + ... + O^{m-1,m}[D] + O^{m-1,m}[D]$$

(C.21)

When $k = 0$, the one simply removed $O^{k-1,k}[D]$ and $O^{k,k+1}[D]$ is not appropriate:

$$O^{12}[D] + O^{23}[D] + .. + O^{k-2,k-1}[D] + O^{k+1,k+2}[D] + ... + O^{m-1,m}[D] + O^{m-1,m}[D]$$

(C.22)

The correct one is the following:

$O^{12}[D] + O^{23}[D] + .. + O^{k-2,k-1}[D] + O^{k-1,k+1}[D] + O^{k+1,k+2}[D] + ... + O^{m-1,m}[D] + O^{m1}[D]$ (C.23)

In this case, we need to introduce a new term $O^{k-1,k+1}[D]$. Hence, when one interval disappears, a new term must be added.

Eq. (C.19) represents the total sum of the coalescent lengths of m stem cells. With this equation, we can rewrite D as follows:

$$D = \frac{1}{2q}\sum_{i=1}^{m}\sum_{j\neq i} k_i k_j = \frac{1}{2q}\left(\sum_{i=1}^{m}\sum_{j=1}^{m} k_i k_j - \sum_{i=1}^{m} {k_i}^2\right)$$

$$= \frac{1}{2q}\left((\textstyle\sum_{i=1}^{m} k_i)^2 - m\frac{1}{m}\sum_{i=1}^{m} {k_i}^2\right) = \frac{1}{2q}\left(n^2 - m\left(var(k_i) + \left(\frac{1}{m}\textstyle\sum_{i=1}^{m} k_i\right)^2\right)\right)$$

$$= \frac{1}{2q}\left(n^2 - m\left(var(k_i) + \left(\frac{n}{m}\right)^2\right)\right) = \frac{1}{2q}\left(n^2 - \frac{n^2}{m} - m \cdot var(k_i)\right) \quad \text{(C.24)}$$

From this, we have

$$D \leq \frac{1}{2q} n^2 \left(1 - \frac{1}{m}\right) \quad \text{(C.25)}$$

This quantity reaches the maximum when m stem cells are sampled at equal intervals. The equality holds, if n can be divided by m and the sampled cells are evenly distributed ($var(k_i) = 0$).

When we sample only two cells ($m = 2$), we have $D = \frac{k(n-k)}{q}$ as derived in Iwasa et al. (2023). The maximum value of this is approximately $D \approx \frac{1}{4q} n^2$, when sampled cells are positioned on the opposite side of the branch $k = \frac{n}{2}$.

When we sample all n stem cells in the shoot apical meristem, we obtain the total coalescent length by simply setting $m = n$ and $k_1 = k_2 = ... = k_m = 1$. Eq. (C.25) becomes as follows:

$$PD = D = \frac{1}{2q}(n^2 - n) = \frac{n(n-1)}{2q} \quad \text{(C.26)}$$

Here, we note that D is, in fact, phylogenetic diversity PD in the text. The ratio of the total coalescent length of the whole stem cells to the maximum coalescent length when only two cells are sampled is

$$\frac{PD(all\ cells)}{max_k D} = \frac{\frac{n(n-1)}{2q}}{\frac{1}{4q}n^2} = \frac{2(n-1)}{n} \tag{C.27}$$

which is slightly less than 2.

We can consider the value of D averaged over k $(k = 1,2,\ldots,m)$. This is the mean pairwise phylogenetic distance, denoted as $\bar{D}$ in the main text. It is calculated as follows:

$$\bar{D} = \frac{1}{n}\sum_{k=1}^{n} D = \frac{1}{n}\sum_{k=1}^{n} \frac{k(n-k)}{q} = \frac{n^2-1}{6q} \tag{C.28}$$

Refer to Iwasa et al. (2023). The ratio of phylogenetic diversity (the total coalescent length of all stem cells) to pairwise phylogenetic distance (the maximum coalescent length when only two cells are sampled is

$$\frac{PD(all\ cells)}{\bar{D}} = \frac{\frac{n(n-1)}{2q}}{\frac{1}{6q}(n+1)(n-1)} = \frac{3n}{n+1} \tag{C.29}$$

which is slightly less than 3.

We note that some of these formulas are similar to those in the classic coalescent theory. However, the model in this paper has a structure of a strong geographic structure (circular stepping-stone model), while the classic coalescent model is about the population without geographic structure.

In summary, the phylogenetic distance of all stem cells in the SAM is approximately three times larger than the mean pairwise phylogenetic distance and about two times larger than the maximum phylogenetic distance between two stem cells.

Part D

Three indexes applied to a branching tree

To illustrate that different indexes capture different aspects of within-individual genetic variation, we consider a case with a simple structure of bifurcating tree analyzed by Tomimoto et al. (2023). We show that these indexes depend on the number of stem cells within SAM.

We consider a tree with a trunk and many branches, which having n stem cells a branch (or trunk) (Tomimoto et al. (2023) the number of stem cells by α). As a branch elongates, stem cells form separate lineages, if the stem cells leave their successor stem cells without failure (no somatic genetic drift). Let d_o be the genetic distance for one step of branch elongation. Lineages of n stem cells initially having the same genome accumulate somatic mutations independently.

To consider the branching architecture, we adopt a model first proposed by Honda (1971). Trees start from a trunk growing from the ground, after elongation by d_o, they form two branches. The number of branch tips double in each step. After four steps of bifurcation, the tree have $16(= 2^4)$ branch tips, as illustrated in Fig. S1(A). Each branch tip has one reproductive organ attached, but let's consider the case we sampled one seed from each of 16 tips.

When a branch (or trunk) bifurcates, two daughter branches are formed: a main branch and a lateral branch. The SAM of the main branch inherits the same n stem cells as in the SAM of the original branch. In contrast, the SAM of the lateral branch is formed from copies of a single stem cells in the SAM of the original branch. Consequently, just after the formation of a new lateral branch, the genetic difference

between stem cells in the SAM disappears, while the same genetic variation is maintained in the main branch. In Figure S1(B), we illustrated the case where $n = 3$, in which three parallel lines indicates three stem cell lines within the SAM of the branch.

Now we consider three indexes in the current paper. We sample a single seeds from each of the 16 branch tips. The index becomes as follows:

[1] Mean phylogenetic distance: $\overline{D}$

$\overline{D}$ is the same as $\bar{Z}$ in Tomimoto et al. Please refer to Tomimoto et al. for the calculation procedure. Refer to Tomimoto et al. (2023) for the details. $\overline{D}$ increases with n, but the rate of increase slows down for large n.

[2] Phylogenetic distance PD:

PD stands for the total length of the lineage tree drawn by the cell lineage. For $n = 1$, the total length of the branches excluding the trunk is $30d_o$. because 2+4+8+16=30. For $n > 1$, the total length including the trunk multiplied by the number of stem cells is $30nd_0$. PD increases with n in direct proportionality.

[3] Parent-offspring distance: D_{PO}

The distance from the root to the tip of each branch is 5, making the average distance to all branch tips also 5. Hence, we have $D_{PO} = 5d_o$

The result is illustrated in Figure S1. Horizontal axis indicates n, the number of stem cells in the SAM.

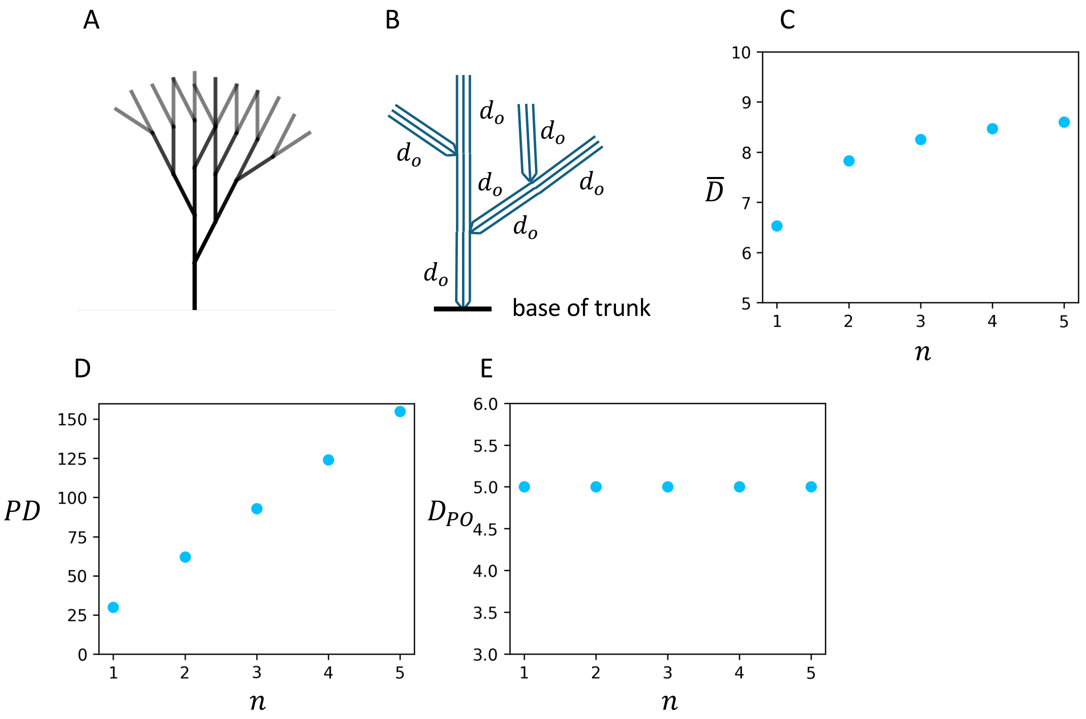

Figure S1. Three indices were calculated on a tree of branching architecture with 16 reproductive organs at each tip of branches. (A) Branching architecture. (B) Three parallel lines are cell lineages. Here we illustrate the case with three stem cells in the SAM ($n = 3$). (C) Mean pairwise phylogenetic distance. Horizontal axis is the number of stem cells: $\bar{D}$. (D) Phylogenetic diversity: PD. (E) Parent-offspring distances: D_{PO}.

Reference:

Honda, H. 1971. Description of the form of trees by the parameters of the tree-like body: effects of the branching angle and the branch length on the shape of the tree-like body. *Journal of Theoretical Biology* 31: 331–338. doi:10.1016/0022-5193(71)90191-3

Part E

Selective effects of genetic diversity in offspring

The genetic variation of phenotypic traits is an indispensable element of evolutionary adaptation. However, mutation also produces maladaptive or nonfunctional genes. We may ask whether having a higher rate of mutation is advantageous in the evolution and whether the tissue structure favoring somatic mutations could be selected for because of its contribution to enhanced genetic diversity. In the following subsection, we briefly summarize major theoretical ideas on the evolution of mutation rate in the population genetics.

(i) Constant and uniform environment

Theoretical study of the evolution of mutation rate includes papers on the evolution of genetic systems (Karlin 1975; Karlin & McGregor 1974). In the simplest setting of the model, there is one selected locus that affects fitness and a second modifier locus that specifies the mutation rate at the selected locus. The modifier may not directly contribute to fitness (a neutral modifier), but it evolves through its impact on the selected locus. In an infinitely large population of haploid organisms, if the allele fitness does not change over time, natural selection causes the first locus to converge to the state of the highest fitness. The population comes to have alleles at a local peak of the fitness landscape. Then the mutations become harmful because they change the optimal allele to a different allele with lower fitness. Hence, the mutation rate evolves to zero. This argument was extended to various situations with nonlinear fitness effects, such as diploid with dominance between alleles, multiple loci with

epistasis, and phenotypic switching (Liberman et al. 2011; Altenberg et al. 2017).

(ii) Temporally fluctuating environments

Under temporally changing environments, alleles advantageous in the current generation may become disadvantageous, while those that will be advantageous later are currently disfavored and rare. The mutation creates promising alleles from those that are currently favored and abundant. A positive mutation rate evolves in a cyclical environment (Ishii et al. 1989), but not in randomly changing environments. This result can be intuitively understood by decomposing the environmental fluctuation into a sum of multiple sinusoidal components with varying periods, provided that selection is not very strong. In an environment fluctuating with a single sinusoidal component, the mutation rate to evolve is inversely proportional to the period. Hence, a high mutation rate evolves under fluctuation with a short period, while a low mutation rate evolves under fluctuation of a long period. On the other hand, the impact of environmental fluctuations on the mutation rate modifier gene and the population mean fitness is weaker for components with short periods (fast fluctuations) than for those with long periods (slow fluctuations). This phenomenon is known as "low-pass filter effect," reported earlier in the theory of recombination rate evolution under fluctuating environments (Sasaki & Iwasa 1987). Furthermore, random fluctuations contain long-period components more than short-period components, as indicated by the monotonic decrease in the power spectrum with the frequency. Consequently, random fluctuations do not make a positive mutation rate evolve, unless selection intensity is very strong.

However, high mutation rates can evolve under frequency-dependent

selection. In the host-pathogen and herbivore-plant coevolution with genotype-specific attacking rates, the fitness is likely to be frequency dependent and a high mutation rate evolves (Haraguchi & Sasaki 1996, 1997; Sasaki & Haraguchi 2000).

(iii) Spatial heterogeneity and severe sib competition.

Another mechanism promoting the evolution of a positive mutation rate considers sib competition. This logic was originally proposed as a mechanism for the evolution of sexual reproduction with recombination (Williams 1975), and was later formalized in a mathematical model (Maynard Smith 1978; Douge & Iwasa 2017a, 2017b), where an element of spatial heterogeneity was incorporated, as a concept known as the "tangled bank" hypothesis (Bell 1982).

Suppose that the habitat consists of many patches and that the environmental condition changes between patches and between generations. Even if a parent's allele is well adapted in the current generation, it may not be adapted in the next generation. If competition is very intense, the best-adapted individuals among the seeds that landed on the same patch take over the patch. The advantage of producing many offspring of the same genotype is much reduced if multiple offspring of the same parent land on the same patch and engage in severe sib competition. In such a situation, a parent with a high mutation rate receives an advantage by producing genetically more diverse offspring.

This scenario seems plausible for many trees that have a limited range of seed dispersal and produce a large number of seeds, leading to intense sib competition. Many trees that rely on gravity-dispersal, such as those producing acorns, have a limited seed dispersal range. However, the effect might be weaker for trees producing

seeds with a long dispersal distance, such as those carried by ants or other animals using elaiosomes, as well as small seeded species like maples and other gap adapted species.

(iv) Mutation enhancing mechanisms

In special occasions, organisms adopt high mutation rates (Metzgar & Willis 2000). In bacteria, a high mutation rate is induced in response to risky environmental conditions, as exhibited by the SOS mechanism (Rosenberg et al. 1998). Other organisms, such as parasite worms like trypanosomes, have genes for surface proteins that allow them to escape host immune responses.

From these observations, we must conclude that a higher rate of mutations in general is disadvantageous, except for specific mechanisms for coping with host-pathogen interaction. An observed positive mutation rate is a result of a large cost accompanied by further reductions in error rates during genome replication and damage repair. A small positive rate of mutation produces genetic variation that forms the basis for the adaptation to environmental changes (Lynch 2010, 2011).

Part F

Difference between $\overline{D}$ **and** PD

Both $\overline{D}$ and PD measure genetic diversity among offspring (or fruits) of a single individual tree. However, they differ in the aspects focusing on the diversity between organs. Since PD counts the number of different genotypes among all the offspring sampled, PD regards rare genotypes as equally important as common genotype. In contrast, $\overline{D}$ count the genetic differences between randomly sampled pair of fruits. As a result, PD regards the branches located in the marginal portion of a tree equally important as those in the central portion, while $\overline{D}$ places more weight on branches (or trunks) in the central portion than those in the marginal position, as shown in Fig. 2.

These two indexes may differ their usefulness in measuring the fitness effects to the parent. To illustrate this, we consider the situation in which the suitability of $\overline{D}$ and PD may depend on the seed survivorship, germination success, or seedling survivorship.

Suppose that the local competition is very intense and only one offspring can survive due to the severe sib-competition. Then only the genotype with the highest fitness can survive and all the others die. Assume that the fitness of a genotype is randomly chosen and the fitness of two genotypes is independent of their sequence similarity. When at least one offspring of the highest fitness can survive, the parent enjoys the same reproductive success as when many seeds with the highest fitness survive. In such a case, the reproductive success of the parental tree depends whether it can leave at least on seed (offspring) of the highest fitness. Then, the number of

offspring of different genotypes determines the fitness of the parent, but having many offspring of the same genotype does not contribute to the success of the parent.

We consider two situations: in situation A, three reproductive organs (i.e., fruits), X, Y, Z which are genetically distant from each other; but both X and Y are much larger in size than Z -- say X, Y, and Z produce 1000 seeds, 1000 seeds, and 10 seeds;. In contrast, in situation B, reproductive organs X, Y, Z are equal in size and each produces 670 seeds. Note that total number of seeds is the same (2010) between the two.

As explained in the text, PD is independent of relative size of reproductive organs. It predicts the same value in situation A and in situation B. In contrast, $\overline{D}$ would predict a larger value for situation B than for situation A, because $\overline{D}$ regards the contribution of Z smaller than the other two. We ask which of the two indexes can predict the fitness of the parent tree?

Now we consider the fitness of the parental tree. Suppose that all the seeds can survive and produce the next generation. In both situations A and B, all three organs X, Y, and Z leave at least one surviving offspring. In this case, PD predicts that the fitness of the tree is the same between A and B. This is a correct prediction. In contrast, $\overline{D}$ failes because it predicts a higher fitness for a tree in situation B and another in situation A fails because it.

The same conclusion holds if the survivorship of seeds is high, say 50%. Still, all three reproductive organs X, Y, and Z produce one or more surviving offspring and trees in situations A and B give the same fitness.

However, the conclusion is different if the survivorship and germination

success are small, and only 2% of the seeds can germinate and grow to the adult in the next generation successfully. Then in situation B, all of X, Y, Z leaves one or more offspring (because $670 * 0.02 = 13.4 \gg 1$). In contrast in situation A, only X and Y can leave one or more offspring (because $1000 * 0.02 = 20 \gg 1$), but Z is likely to leave no surviving offspring (because $10 * 0.02 = 0.2 \ll 1$). Hence, the fitness is high in situation B than in situation A, which is correctly predicted by D but not by PD.
