# Supplementary material for: Genetic diversity within a tree and alternative indexes for different evolutionary effects
Source: Quant Plant Biol. 2024 Dec 6;5:e11. doi: 10.1017/qpb.2024.9 (PMC11706689; doi:10.1017/qpb.2024.9)
Supplement: Peer review history

# Editor report: Recommendation: Genetic diversity within a tree and alternative indexes for different evolutionary effects — R0/PR2

**DOI:** 10.1017/qpb.2024.9.pr2

**Affiliations:** Biology, University of York, United Kingdom of Great Britain and Northern Ireland

Thank you for your submission. Both reviewers commented on the work’s novelty and interest to QPB readership. Pay careful attention to the suggested clarifications that both reviewers suggest as their first major suggestions.

# Editor report: Decision: Genetic diversity within a tree and alternative indexes for different evolutionary effects — R0/PR3

**DOI:** 10.1017/qpb.2024.9.pr3

**Affiliations:** RDP, ENS de Lyon, France

No accompanying comment.

# Editor report: Recommendation: Genetic diversity within a tree and alternative indexes for different evolutionary effects — R1/PR5

**DOI:** 10.1017/qpb.2024.9.pr5

**Affiliations:** Biology, University of York, United Kingdom of Great Britain and Northern Ireland

No accompanying comment.

# Editor report: Decision: Genetic diversity within a tree and alternative indexes for different evolutionary effects — R1/PR6

**DOI:** 10.1017/qpb.2024.9.pr6

**Affiliations:** RDP, ENS de Lyon, France

No accompanying comment.
